# Supplementary material for: Defining Conservation Units in a Highly Diverse Species: A Case on Arctic Charr
Source: Evol Appl. 2025 Dec 28;19(1):e70190. doi: 10.1111/eva.70190 (PMC12745347; doi:10.1111/eva.70190)
Supplement: Supplementary file 1 — Figure S1: Plots showing the values of two estimators DLK2 and FSTIS estimates across the various K (number of genetic clusters) tested in admixture analysis to determine the most likely number of K. Figure S2: Violin plot of Expected Heterozygosity, Nucleotide Diversity and Observed Heterozygosity ranges for all populations. Black dot represents the mean values. Figure S3: Redundancy analysis plot showing the directions each uncorrelated variables pull in across RDA1 and RDA2. Colours represent each of the individual populations in the dataset. Figure S4: R‐squared importance plots for uncorrelated environmental variables in explaining (A) adaptive variance (N = 1,104 SNPs) and (B) variance of climate associated SNPs used for genetic offset analysis (N = 235 SNPs). Figure S5: Principal component analysis run on the 10 uncorrelated environmental variables used to identify the putatively adaptive SNPs. (A) shows the placements of each lake of origin on PC1 and PC2 while (B) shows the corresponding loadings of each of the environmental variables. Figure S6: EV1 vs EV2 from PCA using SNPs (N = 1087) identified from a redundancy analysis when using admixture proportions to control for neutral structure. Individuals are coloured by lake of origin. Figure S7: EV1 vs EV2 from PCA using SNPs (N = 1104) identified from a redundancy analysis run using population level allele frequencies rather than individual SNP calls. Individuals are coloured by lake of origin. Figure S8: Plot of geographic distribution of populations. Figure S9: Relationship between genetic offset scores and levels of observed heterozygosity. Populations in the same lake of origin (i.e., ecotype pairs) are given the same offset score. Figure S10: Genetic offset scores vs latitude under two RCP scenarios, RCP 4.5 (in blue) and RCP 8.5 (orange). Each point represents a different lake of origin. Figure S11: The relationship between genetic offset and distance to sea when only using populations with five or mo [file EVA-19-e70190-s001.zip › eva70190-sup-0001-FigureS1-S11&TableS1-S13.pdf]

Supplemental Information for:

Defining conservation units in a highly diverse species: A case study identifying priority populations of Arctic charr

Sam Fenton, Colin W Bean, Samuel AM Martin, Samuel J Poultney, Anthony Smith, Elvira de Eyto, Kathryn R Elmer, Colin E Adams

Table of Contents:

**Figure S1**	Page 2
**Figure S2**	Page 3
**Figure S3**	Page 4
**Figure S4**	Page 5
**Figure S5**	Page 6
**Figure S6**	Page 7
**Figure S7**	Page 8
**Figure S8**	Page 9
**Figure S9**	Page 10
**Figure S10**	Page 11
**Figure S11**	Page 12
**Table S1**	separate
**Table S2**	Page 13
**Table S3**	separate
**Table S4**	Page 14
**Table S5**	separate
**Table S6**	Page 14
**Table S7**	Page 15
**Table S8**	separate
**Table S9**	Page 15
**Table S10**	separate
**Table S11**	separate
**Table S12**	Pages 16-17
**Table S13**	Page 17

Figure S1.

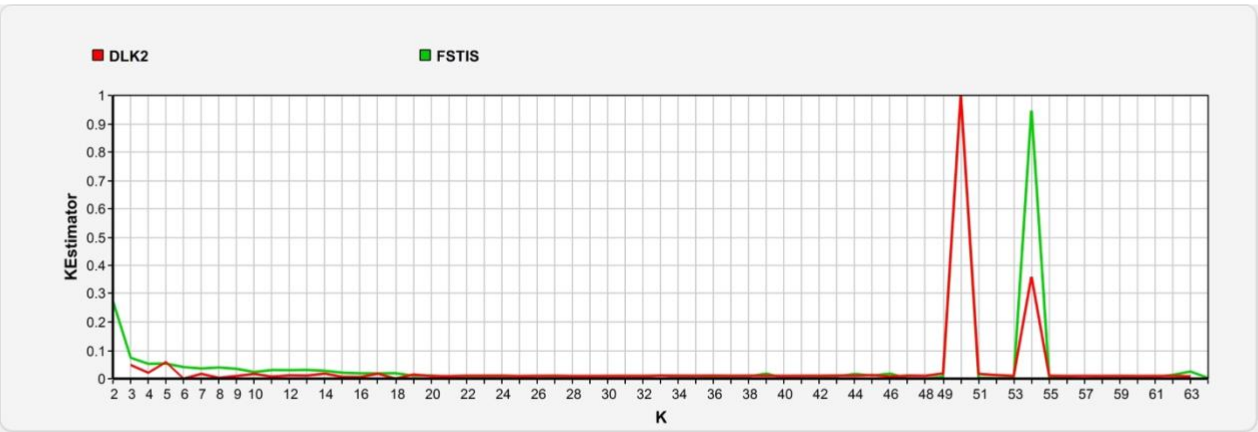

Figure S1. Plots showing the values of two estimators DLK2 and FSTIS estimates across the various K (number of genetic clusters) tested in admixture analysis to determine the most likely number of K. Higher values for each indicator suggest a more probable K value. Detailed explanation of how the estimators are calculated can be found in Wang et al. 2022.

Figure S2.

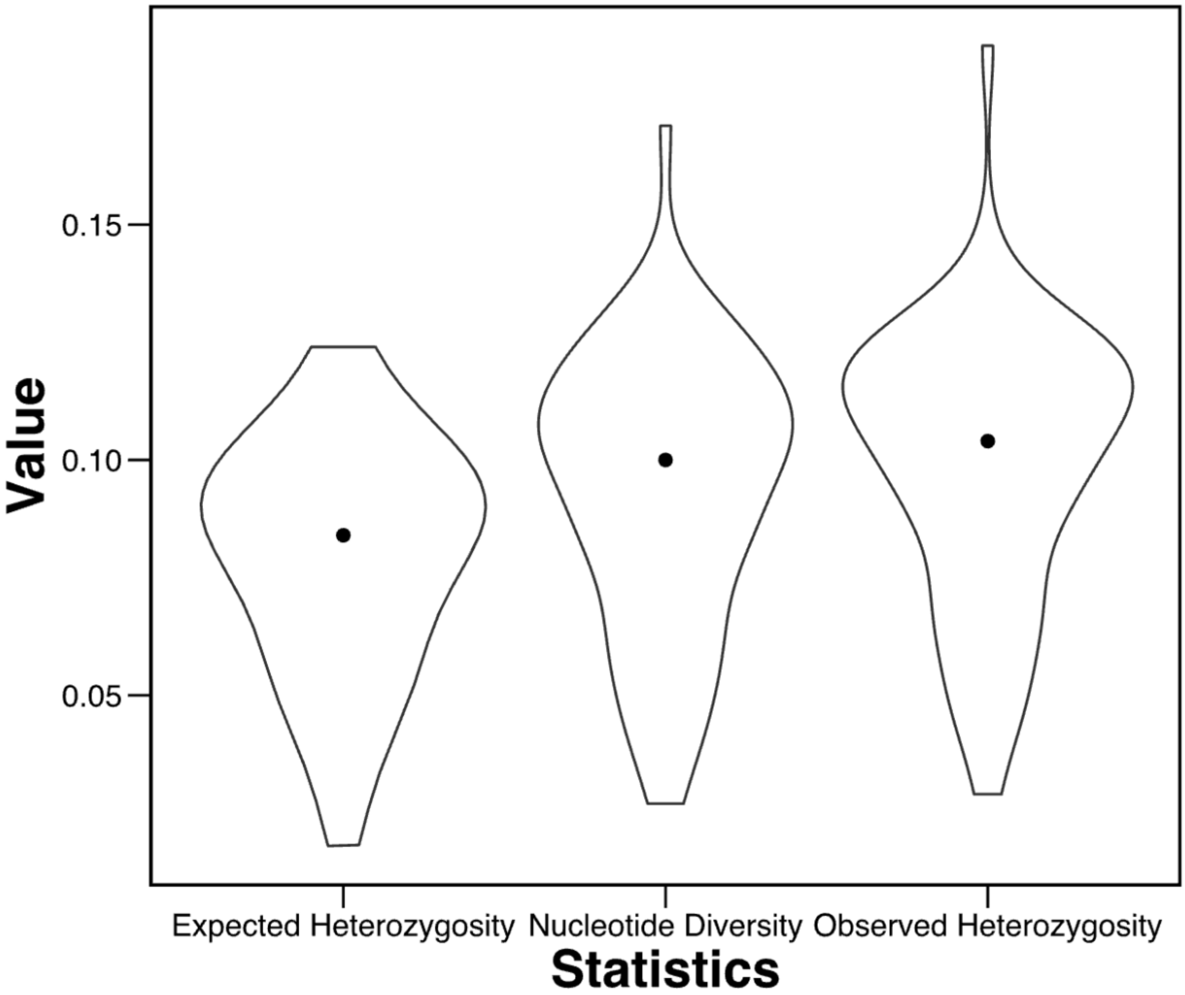

Figure S2. Violin plot of Expected Heterozygosity, Nucleotide Diversity and Observed Heterozygosity ranges for all populations. Black dot represents the mean values.

Figure S3.

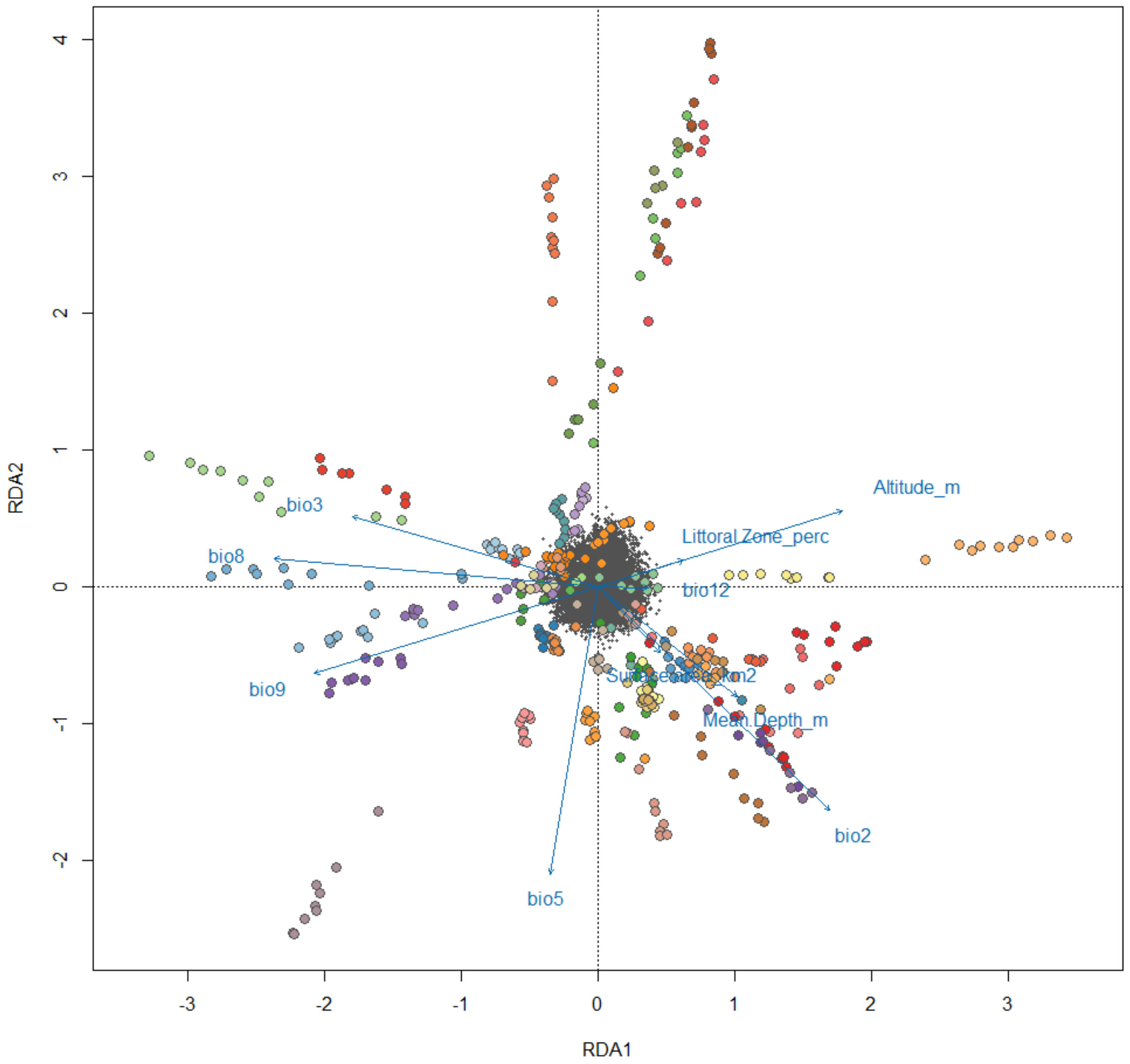

Figure S3. Redundancy analysis plot showing the directions each uncorrelated variables pull in across RDA1 and RDA2. Colours represent each of the individual populations in the dataset.

Figure S4.

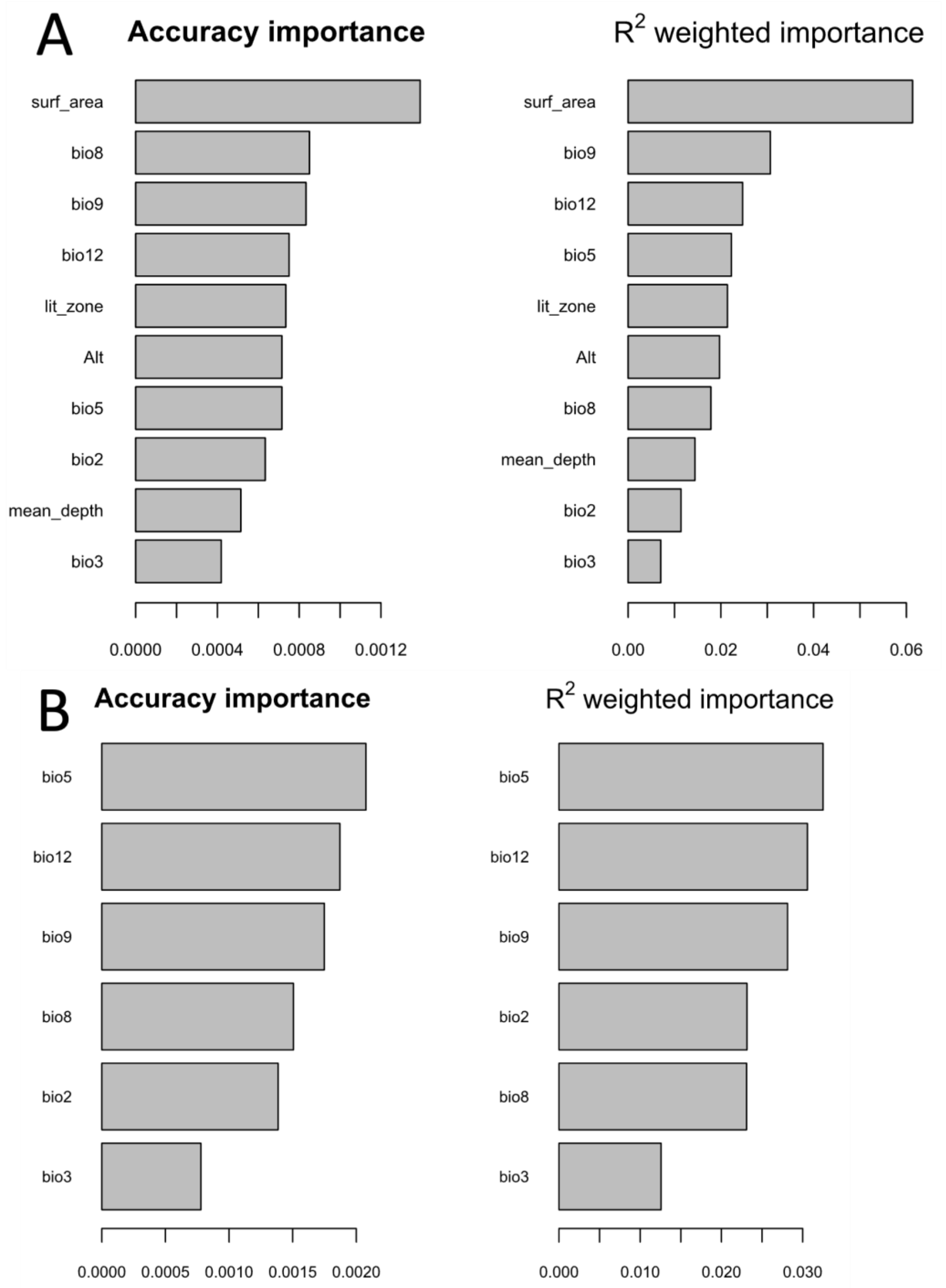

Figure S4. R-squared importance plots for uncorrelated environmental variables in explaining A) adaptive variance (N=1,104 SNPs) and B) variance of climate associated SNPs used for genetic offset analysis (N=235 SNPs).

Figure S5.

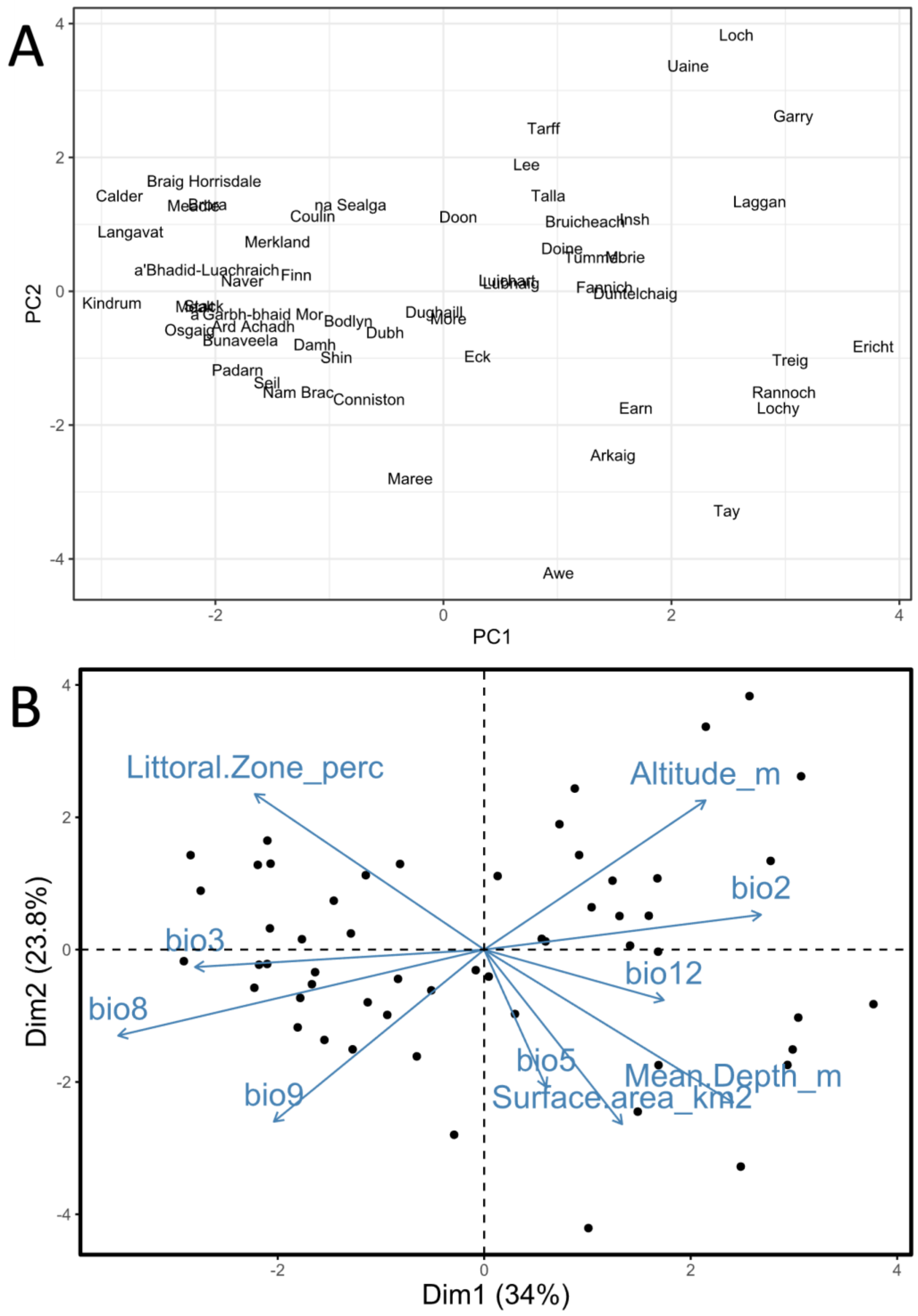

Figure S5. Principal component analysis run on the 10 uncorrelated environmental variables used to identify the putatively adaptive SNPs. A) shows the placements of each lake of origin on PC1 and PC2 while B) shows the corresponding loadings of each of the environmental variables.

Figure S6.

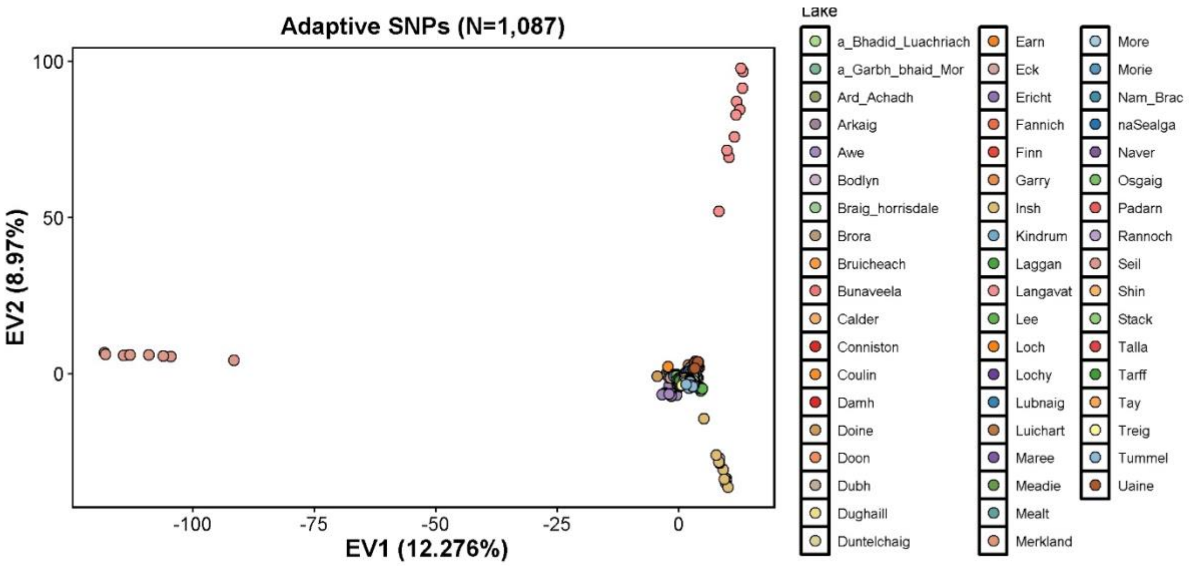

Figure S6. EV1 vs EV2 from PCA using SNPs (N=1087) identified from a redundancy analysis when using admixture proportions to control for neutral structure. Individuals are coloured by lake of origin.

Figure S7.

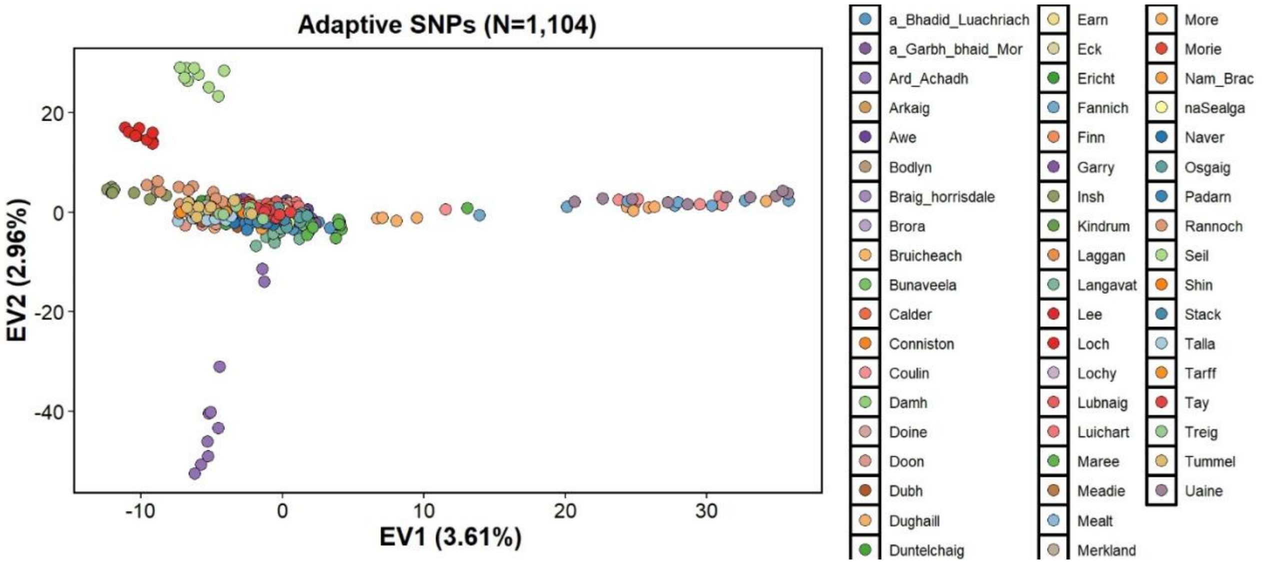

Figure S7. EV1 vs EV2 from PCA using SNPs (N=1104) identified from a redundancy analysis run using population level allele frequencies rather than individual SNP calls. Individuals are coloured by lake of origin.

Figure S8.

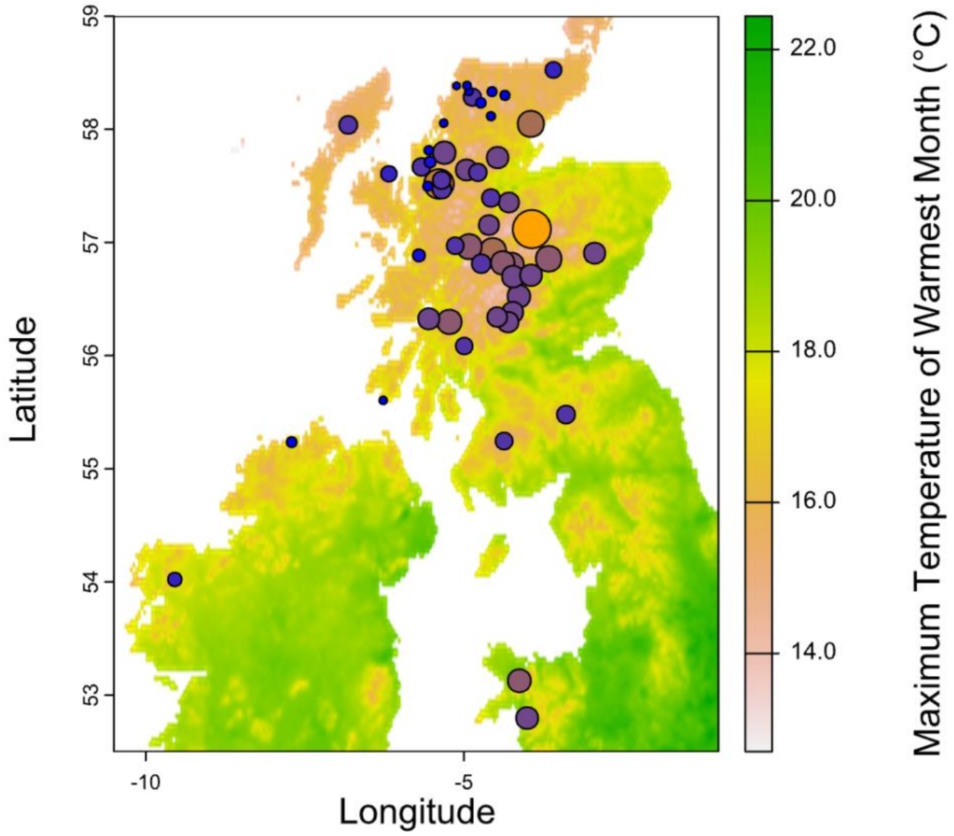

Figure S8. Plot of geographic distribution of populations. Circle sizes and colour reflect genetic offset scores (under the RCP 4.5 scenario) with larger and more orange ones representing a higher offset score. Map of the British Isles is coloured by Bio5 (Maximum temperature of the Warmest Month) readings.

Figure S9.

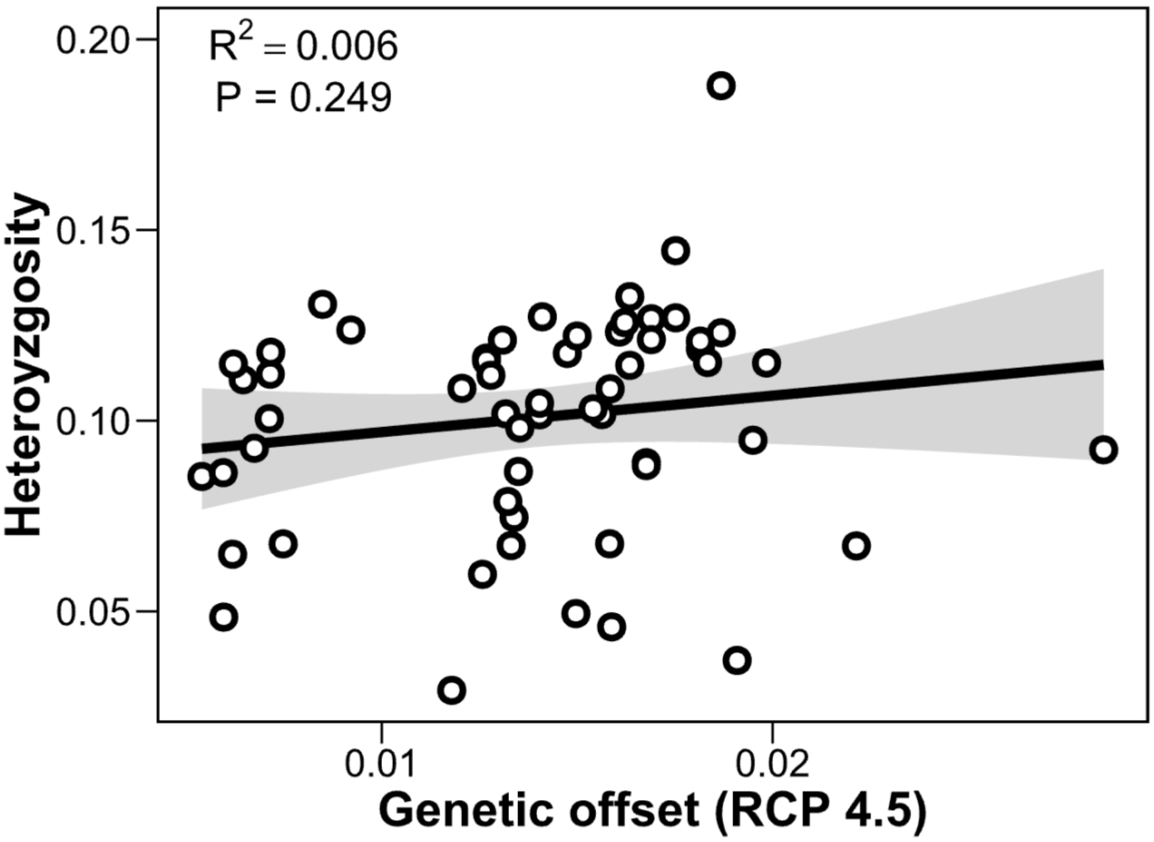

Figure S9. Relationship between genetic offset scores and levels of observed heterozygosity. Populations in the same lake of origin (i.e. ecotype pairs) are given the same offset score.

Figure S10.

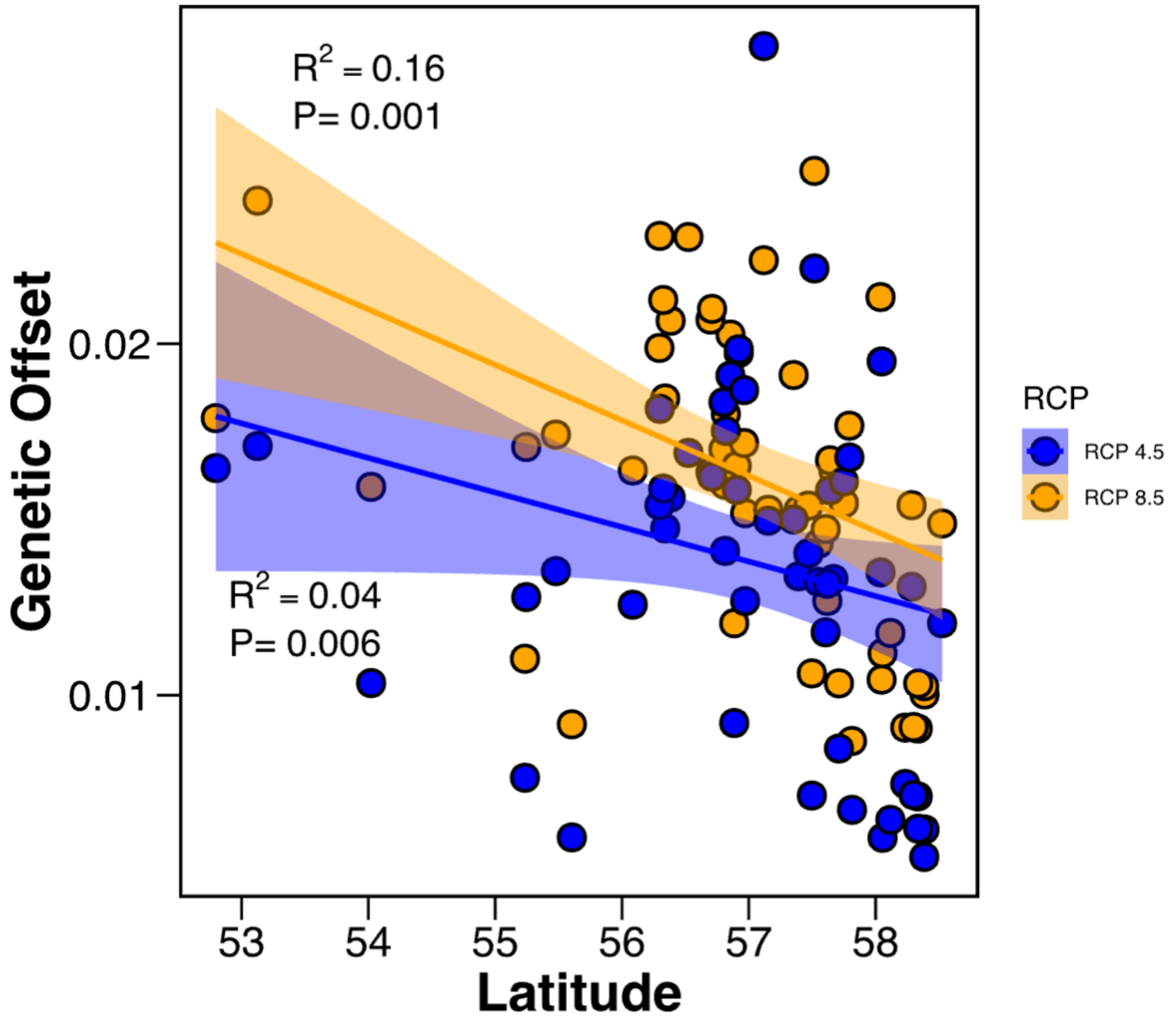

Figure S10. Genetic offset scores vs latitude under two RCP scenarios, RCP 4.5 (in blue) and RCP 8.5 (orange). Each point represents a different lake of origin.

Figure S11:

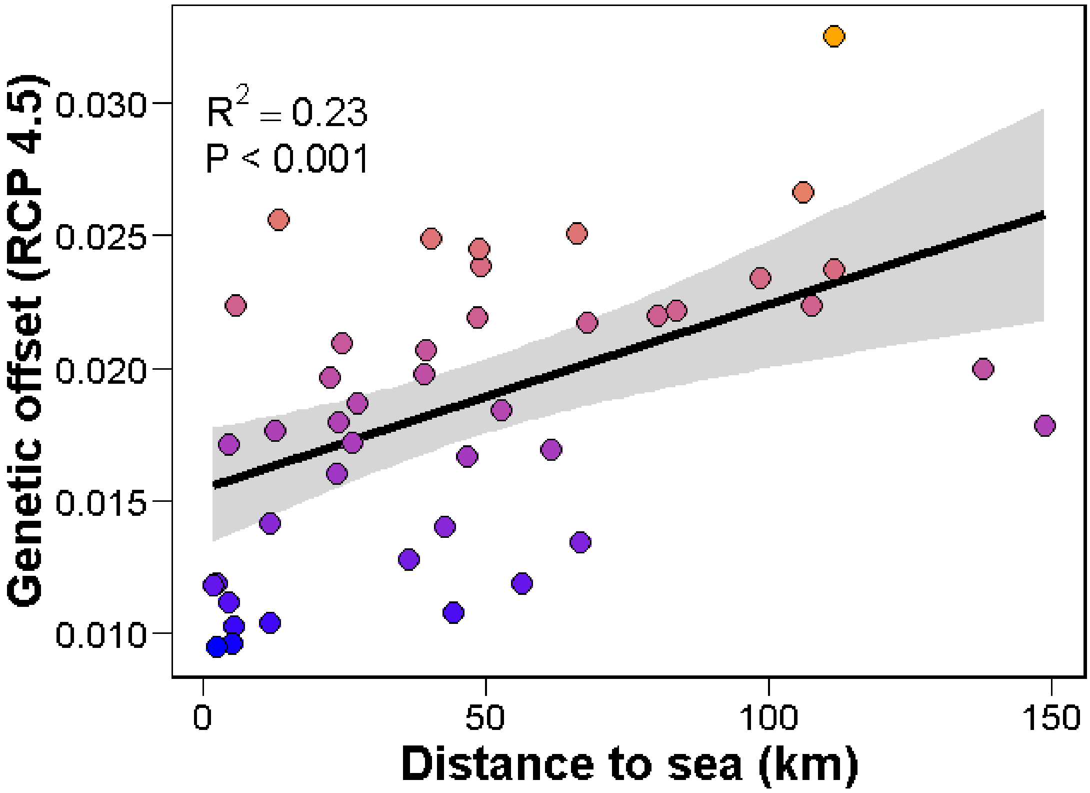

Figure S11: The relationship between genetic offset and distance to sea when only using populations with five or more individuals when calculating genetic offset.

Table S2.

Table S2. List of the 19 climatic variables and 5 bathymetric variables used in the study. Code and full names are provided. Variables in bold are the 10 uncorrelated variables used to identify adaptive SNPs.

Code	Variable
Bio1	Annual mean temperature
**Bio2**	**Mean diurnal range**
**Bio3**	**Isothermality (Bio2/Bio7)**
Bio4	Temperature Seasonality
**Bio5**	**Maximum temperature of the warmest month**
Bio6	Minimum temperature of the coldest month
Bio7	Temperature annual range (Bio5-Bio6)
**Bio8**	**Mean temperature of wettest quarter**
**Bio9**	**Mean temperature of driest quarter**
Bio10	Mean temperature of warmest quarter
Bio11	Mean temperature of coldest quarter
**Bio12**	**Annual precipitation**
Bio13	Precipitation of wettest month
Bio14	Precipitation of driest month
Bio15	Precipitation seaonality
Bio16	Precipitation of wettest quarter
Bio17	Precipitation of driest quarter
Bio18	Precipitation of warmest quarter
Bio19	Precipitation of coldest quarter
**Alt**	**Altitude**
Max.Depth	Maximum lake depth
**Mean.Depth**	**Mean lake depth**
**S.Area**	**Lake surface area**
**Lit.Zone**	**Percentage of the lake that is < 5 metres deep (Littoral zone)**

Table S4.

Table S4. Scoring system used to calculate lake sensitivity scores based on lake surface area, depth, and altitude.

Max depth (m)	Depth sensitivity score	Surface Area (ha)	Surface Area sensitivity score	Altitude (m)	Altitude sensitivity score
<10	4			<10	4
10 to 30	3	<10	3	10 to 50	3
30 to 100	2	10 to 30	2	50 to 200	2
>100	1	>30	1	>200	1

Table S6.

Table S6. Hierarchical AMOVA results showing the variance explained by various groupings including the east-west drainage divide seen in the neighbour-joining tree and hydrometric area of origin.

	Degrees of freedom	Sum squares	Mean squares
Between East-West split	1	7017.08	7017.08
Between Hydrometric Areas within East-West split	30	117095.98	3903.20
Between Populations within Hydrometric Areas	32	68307.97	2134.62
Between Samples within Populations	345	69268.06	200.20
Within Samples	410	108513.12	264.67
Total	819	370202.21	451.02

	Sigma	%
Variation between East-West split	2.54	0.55
Variation between Hydrometric Areas within East-West split	62.39	13.56
Variation between Populations within Hydrometric Areas	162.68	35.36
Variation between Samples within Populations	-32.23	-7.01
Variation within Samples	264.67	57.53
Total variation	460.04	100.00

	Phi
Phi-Samples-total	0.43
Phi-Samples-Populations	-0.14
Phi-Populations-Hydrometric Areas	0.41
Phi-Hydrometric Areas-East West split	0.14
Phi-East West split-total	0.01

Table S7.

Table S7. ANOVA results showing the significance of each constrained axis in the RDA in explaining variance

	DF	Variance	F	Pr (>F)	
RDA1	1	459.2	8.3956	0.001	***
RDA2	1	446.3	8.1611	0.001	***
RDA3	1	387.3	7.0814	0.001	***
RDA4	1	351.8	6.4328	0.001	***
RDA5	1	307.7	5.6268	0.001	***
RDA6	1	292.4	5.3457	0.001	***
RDA7	1	261.8	4.7869	0.001	***
RDA8	1	213.7	3.9076	0.001	***
RDA9	1	183.6	3.3562	0.001	***
RDA10	1	152.1	2.7815	0.001	***
Residual	399	21822.1			

Table S9.

Table S9. Number of putatively adaptive SNPs (N=1,104) each uncorrelated environmental variables was the strongest predictor for in the Redundancy Analysis.

Predictor	Number of SNPs
Surface area	234
Mean depth	70
Littoral zone percentage	35
Altitude	50
Bio2	166
Bio3	156
Bio5	127
Bio8	29
Bio9	166
Bio12	71

Table S12.

Table S12. List of QTLs from Fenton *et al.* 2024b that contained adaptive SNPs we identified. QTL marker names are taken from the mentioned paper. Species of origin and trait the QTL is associated with are also listed. Chromosome, QTL start, and end refer to QTL location when mapped to the *Salvelinus sp.* genome.

QTL marker	Species	Trait	Chromosome	QTl start	QTl end
Cocl_BS_096	*Coregonus clupeaformis*	Body Shape	NC_036838.1	46685809	46685809
Omy_SM_031	*Oncorhynchus mykiss*	Sexual maturation	NC_036838.1	50898901	50898901
Cocl_GR_004	*Coregonus clupeaformis*	Directional change	NC_036839.1	10561411	10561411
Cocl_BS_021	*Coregonus clupeaformis*	Body Shape	NC_036842.1	34066589	34066589
Sal_CF_011	*Salvelinus alpinus*	Condition Factor	NC_036842.1	5751355	88198010
Cocl_DirCh_008	*Coregonus clupeaformis*	Directional change	NC_036842.1	11649948	11649948
Omy_DR_003	*Oncorhynchus mykiss*	Disease Resistance	NC_036842.1	5127503	5127503
Cocl_BS_053	*Coregonus clupeaformis*	Body Shape	NC_036843.1	22695609	22695609
Omy_SM_014	*Oncorhynchus mykiss*	Sexual maturation	NC_036845.1	29414044	29414200
Oki_BL_009	*Oncorhynchus kisutch*	Body Length	NC_036847.1	24097769	32996863
Omy_DR_012	*Oncorhynchus mykiss*	Disease Resistance	NC_036848.1	13363254	13363254
Sal_UTT_004	*Salvelinus alpinus*	Upper temperature tolerance	NC_036848.1	10119484	21971402
Omy_BW_016	*Oncorhynchus mykiss*	Body Weight	NC_036853.1	10667932	17403275
Omy_CF_009	*Oncorhynchus mykiss*	Condition Factor	NC_036853.1	10667932	17403275
Oki_SP_005	*Oncorhynchus kisutch*	Spawning date	NC_036853.1	10667932	17403275
Sna_BS_006	*Salvelinus namaycush*	Body Length	NC_036854.1	3663455	3663455
Sna_BL_133	*Salvelinus namaycush*	Body Length	NC_036855.1	50063281	50063281
Sna_BL_043	*Salvelinus namaycush*	Body Length	NC_036855.1	32096909	32096909
Omy_OSMO_005	*Oncorhynchus mykiss*	Sodium/Chloride concentration 2	NC_036855.1	17674273	17674527
Omy_BW_031	*Oncorhynchus mykiss*	Body Weight	NC_036856.1	18229605	18229605
Omy_SP_018	*Oncorhynchus mykiss*	Spawning date	NC_036856.1	42026512	42027014
Sal_CF_095	*Salvelinus alpinus*	Condition Factor	NC_036861.1	875380	875380
Sal_HS_019	*Salvelinus alpinus*	Head Shape	NC_036861.1	875380	875380
Oki_SP_011	*Oncorhynchus kisutch*	Spawning date	NC_036862.1	27196736	35780323
Sna_BL_221	*Salvelinus namaycush*	Body Length	NC_036863.1	42690182	42690182
Sfo_GR_005	*Salvelinus alpinus*	Growth Rate	NC_036865.1	12786571	12786571
Sal_BW_072	*Salvelinus alpinus*	Body Weight	NC_036866.1	14035337	44737836
Cocl_DirCh_001	*Coregonus clupeaformis*	Directional change	NC_036869.1	19718333	19718333
Cocl_DirCh_005	*Coregonus clupeaformis*	Directional change	NC_036870.1	5780669	5780669
Oki_SM_002	*Oncorhynchus kisutch*	Sexual maturation	NC_036871.1	12958985	12958985

Sna_BS_058	*Salvelinus namaycush*	Body Length	NW_019942665.1	366314	366314
Cocl_BS_169	*Coregonus clupeaformis*	Body Shape	NW_019942665.1	364528	364528
Cocl_BS_167	*Coregonus clupeaformis*	Body Shape	NW_019943451.1	91315	91315
Cocl_BS_101	*Coregonus clupeaformis*	Body Shape	NW_019943516.1	79438	79438
Sal_BL_005	*Salvelinus alpinus*	Body length	NW_019945303.1	61402	61582

Table S13.

Table S13. Correlation matrix between the different metrics of population vulnerability: genetic offset scores, lake sensitivity scores, and observed heterozygosity.

	**Genetic offset**	**Sensitivity score**	**Heterozygosity**
Genetic offset	1.00	-0.40	0.26
Sensitivity score	-0.40	1.00	-0.45
Heterozygosity	0.26	-0.45	1.00
